# Supplementary material for: Saccharomyces cerevisiae as a Toolkit for COP9 Signalosome Research
Source: Biomolecules. 2021 Mar 25;11(4):497. doi: 10.3390/biom11040497 (PMC8065851; doi:10.3390/biom11040497)
Supplement: Supplementary file 1 [file biomolecules-11-00497-s001.zip › biomolecules-1090353-supplementary.pdf]

Supplemental Data for accompanying manuscript entitled:

Saccharomyces cerevisiae as a toolkit for COP9 signalosome research

Dana Harshuk-Shabso[1#], Noam Castel[2#], Ran Israeli[3], Sheri Harari[3] and Elah Pick[1, 2, 3*]

[1]Department of Human Biology, Faculty of Natural Sciences, University of Haifa, Haifa 31905, Israel ; [2]Department of Evolutionary and Environmental Biology, Faculty of Natural Sciences, University of Haifa, Haifa 31905, Israel ; [3]Department of Biology and Environment, Faculty of Natural Sciences, University of Haifa at Oranim, Tivon 36006, Israel

# Shared co-first authorship

*To whom correspondence should be addressed.

This File contains:

Three supplementary figures S1-S6 P. 2-7

Three supplementary Tables S1-S4 p. 8-11

Supplemental Alignments p. 12-22

Supplementary References p. 23

Fig S1

A

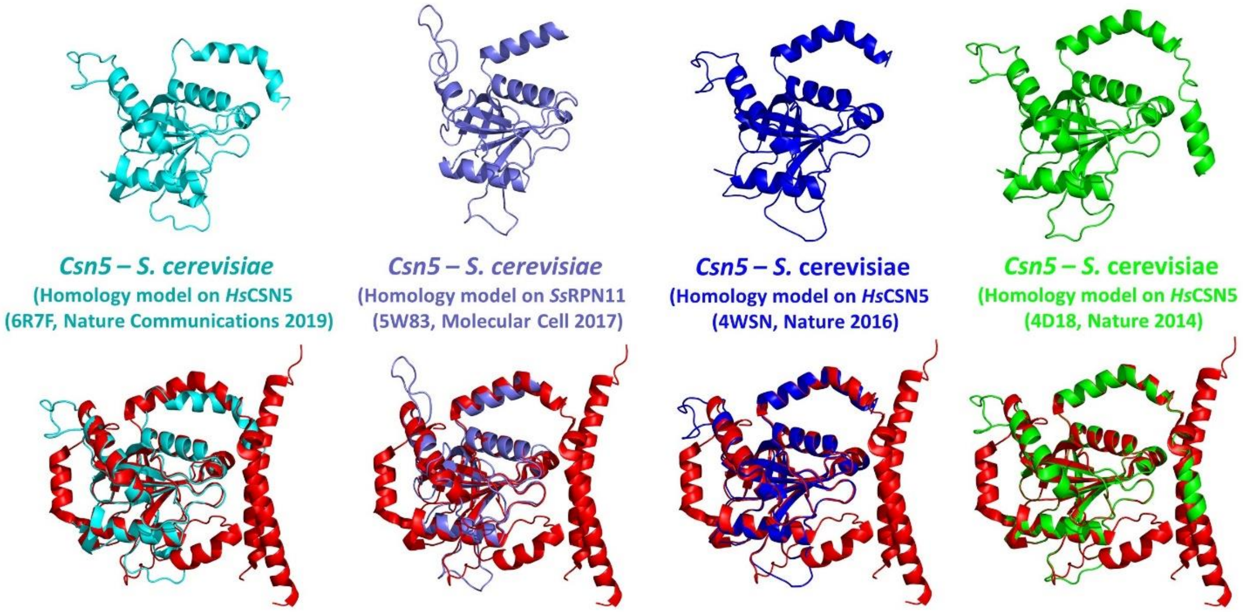

B

S. cerevisiae **CSN5 Homology model based on various CSN5 (*H. sapiens*) structures**

	**4D18 Lingaraju et al. 2014**	**4WSN Cavadini et al. 2016**	**6R7F Faull et al. 2019**	**5W83 Worden et al. 2017**
Sequence identity (%)	39.3%	42.1%	42.1%	27.37%
Coverage (%)	47%	43%	43%	41%
GMQE	0.25	0.21	0.19	0.18
QMEAN	-0.62	-2.09	-3.16	-3.13

Supplemental Figure S1 (for Figure 1C): Structural comparison between *Homo sapiens* and *Saccharomyces cerevisiae* orthologues of CSN5.

(A) *S. cerevisiae* Csn5 homology model comparison suggest that a highly conserved architecture between the *Homo sapiens* (Red) and *Saccharomyces cerevisiae* (other colors) orthologues. CSN5 Homology models were built using the template of four different available CSN5 crystal structures (PDB IDs: 6R7F, 5W83, 4WSN and 4D18), and were superimposed on the *H. sapiens* CSN5 crystal structure (PDB ID: 4D18). **(B)** Query coverage and percentage of identity of the sequences used in A. * References for Figure S1: [1-4]. GMQE (Global Model Quality Estimation) > 0 is an estimator for good accuracy of a model built with that alignment and template structure [5]. The QMEAN (Qualitative Model Energy ANalysis) > (-4) provides reliability estimation for the "degree of nativeness" of the structural features in the model [6].

Fig S2

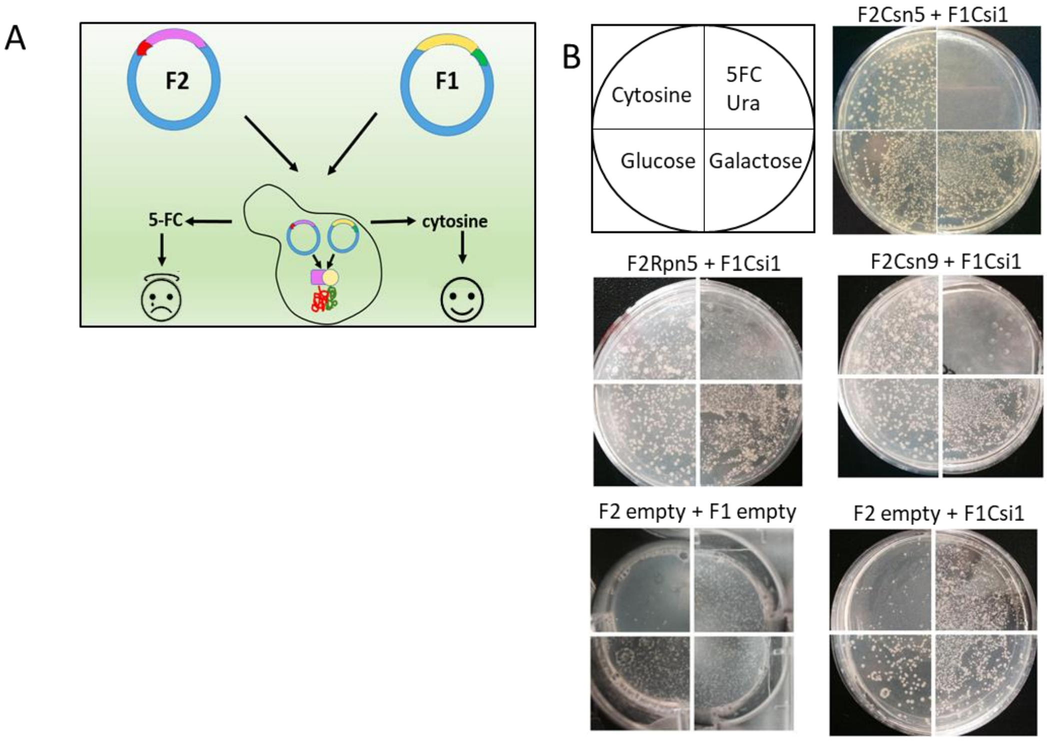

Supplemental Figure S2 (for Figure 2): Evaluating interactions between CSN subunits.

Protein-fragment Complementation Assay (PCA) was approached to detects proximal interactions between CSN subunits. The assay is based on an optimized *S. cerevisiae* cytosine deaminase (FCY1) as a reporter that allows death and survival assay by deaminating cytosine to the essential uracil (survival assay) but also deaminates 5-fluorocytosine (5-FC) to 5-fluorouracil (5-FU), which forms a toxic compound that causes death (death assay). If the candidate proteins interact with each other, the 2 distinct parts of the FCY1 enzyme (A, green and red) are reconstituted and the enzyme becomes active. Accordingly, yeast cells can grow in selection medium lacking uracil, but contain cytosine or no longer grow in medium containing uracil but complemented with 5-FC (B, top and middle). The lack of interaction between the two reporter parts, leads to opposite results (B, bottom).

Fig S3

A

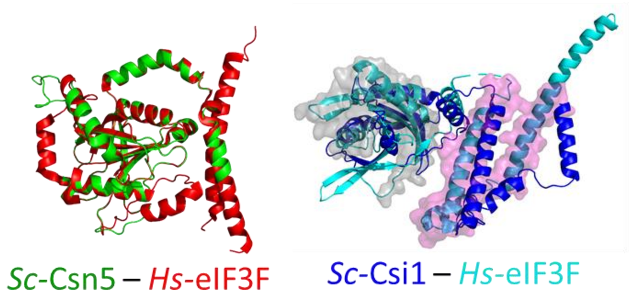

B

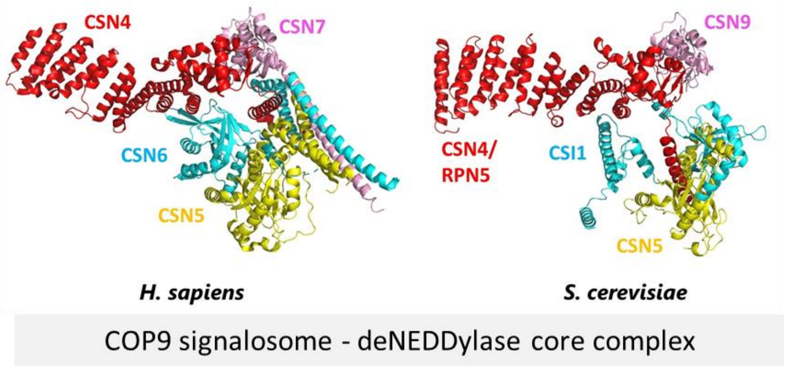

Supplemental Figure 3 (for Figure 2C, D): Superimposition of the *S. cerevisiae* CSN deNEDDylase core complex with the corresponding *H. sapiens* CSN

(A) Superimposition of the *S. cerevisiae* Csn5 (green) and Csi1 (blue) homology models with their corresponding *H. sapiens* CSN5 and CSN6 template structures (Red and cyan respectively, PDB ID 4D18 for Csn5 and eIF3F PDB ID A5T5 for Csi1). Grey and pink surfaces representation is shown for the CSN6/Csi1 MPN and S6CD domains, respectively. (D) Empirical 3D structure of the human and *S. cerevisiae* CSN deNEDDylase core complexes. *S. cerevisiae* homology models were built using Swiss-Model for Csn5, CSN4/Rpn5 and CSN7/Csn9, or Phyre[2] for Csi1 (using eIF3F as a template). Note that residues that are not aligned with the sequence of the template are not shown in this predicted comparative model.

Fig S4

A

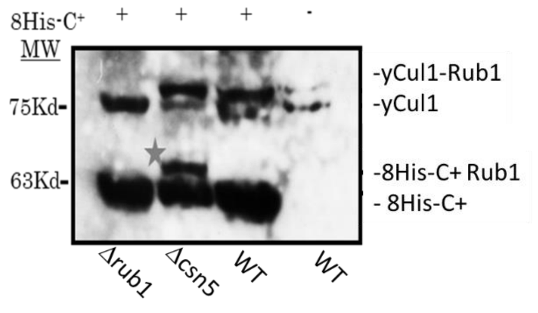

B

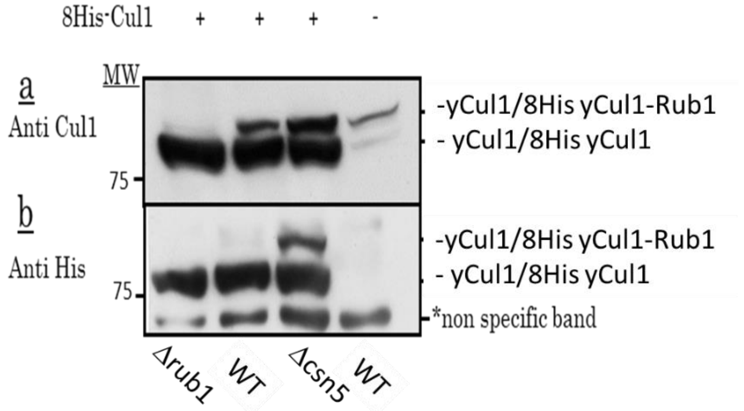

Supplemental Figure 4 (for Figure 3).

(A) **Neddylation status of the 8His-C548 truncation mutant.** Total cell extract of logarithmic wildtype, *Δcsn5* and *Δnedd8* mutant strains, expressing 8His-C548 used for immunoblotting with anti-yCul1 antibody.

(B) **Neddylation status of ectopic 8His-Cul1.** Total cell extract of logarithmic wildtype, *Δcsn5* and *Δnedd8* mutant strains, expressing 8His-yCul1 (FL815) approached for immunoblotting with (a) anti-yCul1 antibody and (b) anti-His antibody.

Fig S5

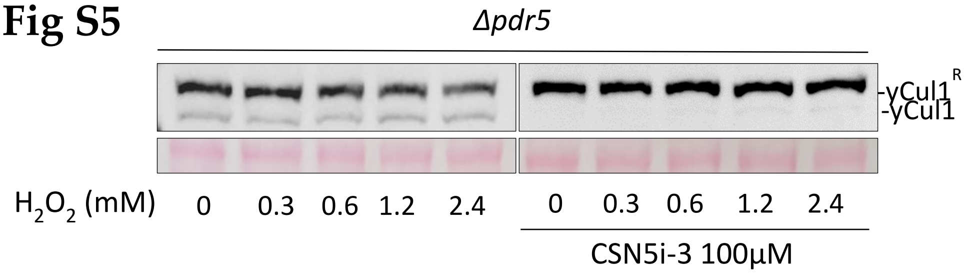

Supplemental Figure 5 (for Figure 6): Cullin neddylation status in *Δpdr5* cells is not affected by H_2O_2.

Logarithmic *Δpdr5* cells were treated with H_2O_2 as indicated. With or without the addition of 100 μM CSN5i-3. Representative immunoblots of anti-yCul1 (7.5% SDS PAGE) show yCul1neddylaiton status. The results show that unlike the wildtype strain [7], the addition of H_2O_2 has neither led to alterations in cullin neddylation status (left), nor inhibited CSN activity (right).

Fig S6

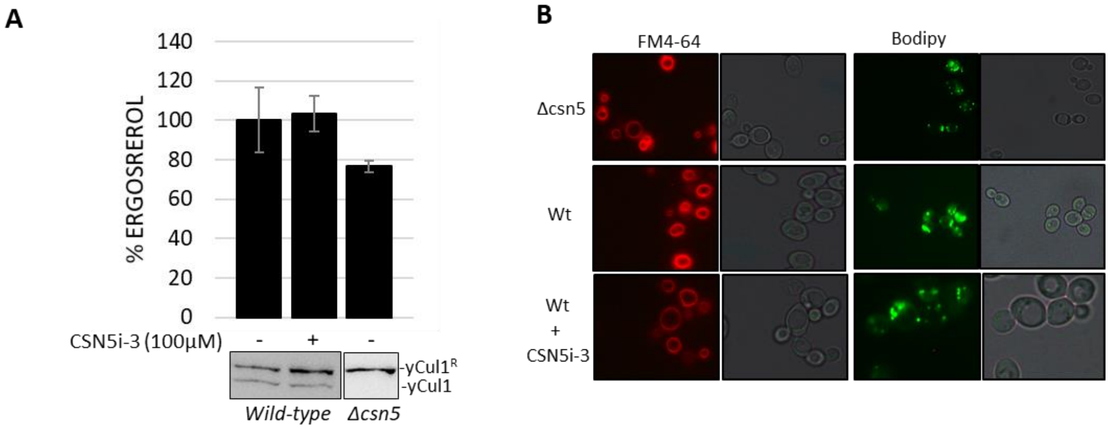

Supplemental Figure 6 (for Figure 6): Testing the phenotype of *S. cerevisiae* cells pre-treated with CSN5i-3.

S. cerevisiae wildtype and *Δcsn5* mutant cells were diluted to 0.5 OD_{600} and grown 4 hours to the log phase. Samples were treated with the CSN5i-3 inhibitors for 2 hours. The percentage of ergosterol upon treatment with the inhibitor (200 µM) in each strain was determined according to Sinha et al. 2020 [8] (A, top). Inhibition of yCul1 deneddylation by the inhibitor is shown below the graph (A, bottom). The same culture was used to evaluate if the Csn5i-3 treatment confer to wildtype cells phenotypes of *Δcsn5* mutant cells (B). Accordingly, vacuole morphology was assessed by FM46-4 (16 µM) and natural lipids by treatment with BODIPY493/503 (1 µM). No changes in wildtype morphology was observed. Intensities were observed with a NIKON Eclipse E600 fluorescent microscope. Representative images are shown.

Table S1 (Plasmids)

Number	Name	Description	Source
EP53	Empty vector	(Leu) Yeplac181 including ADH1*p* promoter and terminator	[9]
EP115	Rub1-GG	(Leu) Yeplac181, ADH*p*, RGS-Rub1ΔN77	This study
EP153	8HIS-Ub	(Leu) Yeplac181, ADH*p*, RGS-8HIS-Ub (k0)	[10]
EP172	ΔN623	(Leu) Yeplac181, ADH1*p*, RGS-8His -Cul1 (623-815aa)	This study
EP181	6His-Flag-Rub1	(Leu) Yeplac181, ADH*p*, 6His-Flag-Rub1(intron-free)	This study
EP233	GFP-Rub1	(Ura) pYES2, GAL1*p*, ADH*p*, GFP-Rub1	This study
EP237	8His-yCul1	(Leu) Yeplac181, ADHp, Cul1 (1-815aa) (Full length)	This study
Ep238	8His-C^+	(Leu) Yeplac181, ADH*p*, Cul1 (267-815aa)	This study
EP247	ΔN438	(Leu) Yeplac181, ADH*p*, 8HIS- Cul1 (438-815aa)	This study
EP248	ΔN522	(Leu) Yeplac181, ADH*p*, 8His-Cul1 (522-815aa) under	This study
EP250	PCA F1	(His) GAL*p*, F1 yeast cytosine deaminase	
EP251	PCA F2	(Leu) GAL*p*, F2 yeast cytosine deaminase	
EP252	PCA F2 CSN5	(Leu) GAL*p*, F2 yeast cytosine deaminase *CSN5*	This study
EP258	ADH-GFP-Nedd8	(Ura) ADHp, GFP-Nedd8	This study
EP259	PCA F2 CSN9	(Leu) GAL*p*, F2 yeast cytosine deaminase *CSN9*	This study
EP260	PCA F1 CSI1	(His) GAL*p*, F1 yeast cytosine deaminase *CSI1*	This study

Table S2 (*S. cerevisiae* strains)

Name	Strains	Genotype	Source
**YP65**	wildtype	BY4741; (his3ko; leu2ko; met15ko; ura3ko); Mat a	Open Biosystems
**YP193**	wildtype	W303; (Ura3-52,Lys2-807,ade2-101,trp1-^36, his3-^200, leu2-^1); Mat a	[8]
**YP333**	*Δcsn5*	W303; ΔYDL216C::G418, Mat a	[8]
**YP146**	*Δcsn5*	**Dcsn5** (*Dcsn5* BY4742; (his3ko1; leu2ko0; lys2ko0; ura3ko0;): YDL216C::G418; mat alpha	Open Biosystems
**YP142**	*Δrub1*	BY4742; YDR139C::G418; mat alpha	Open Biosystems
**YP67**	*Δpdr5*	W303; pdr5::hisG, Mat a	
**YP 147**	*Δcsn5*	BY4741; his3ko; leu2ko; met15ko; ura3ko YDL216C::G418	Open Biosystems
**YP 331**	*Δ rub1*	W303; YDR139C::G418, Mat a	[8]
**YP 421**	*Δcsn5 Δnedd8*	BY4741; YDL216C::hisMX6 YDR139c:kanMX4 Mat a	This study
**YP 485**	CSN9-MYC Csn10-CBP	CSN9 and CSN10 tagged with MYC and CBP respectively	[9]
**YP480**	PCA parental strain (*Δ fcy1*)	BY4741; YPR062W: kanMX4 Mat a	Open Biosystems

Table S3 (DNA primers)

3.1.1 Primers

Num.	Description	Sequence
**Pr48**	8His-Cul1 NTD (forward) designed for ligation with plasmid EP153	GATCGCATCACCATCACCATCACCATCACGGATCCGAGACTCTGCCTAGATCT
**Pr49**	8His-C$^+$ NTD (forward) designed for ligation with plasmid EP153	GATCGCATCACCATCACCATCACCATCACGGATCCACAATATATTGGGATGATCAT
**Pr50**	ΔN438 (forward) designed for ligation with plasmid EP153	GATCGCATCACCATCACCATCACCATCACGGATCCCTAGCTAAGTACAGTGATAT
**Pr51**	ΔN522 NTD (forward) designed for ligation with plasmid EP153	GATCGCATCACCATCACCATCACCATCACGGATCCTTTCAAGATATTAGACTTTCC
**Pr52**	ΔN623 NTD (forward) designed for ligation with plasmid EP153	GATCGCATCACCATCACCATCACCATCACGGATCCTTCAACTTTACGGTAACACT
**Pr54**	Cul1 (reverse) designed for ligation with plasmid EP153	GCTTATTTAGAAGTGTCATCTAGAGGATCCCTGCAGTTAAGCAAGGTAAGCATACG
**Pr55**	GFP-Nedd8 (forward) designed for ligation with plasmid EP222	ATTACACATGGCATGGATGAACTATACAAAATTGTTAAAGTGAAGACACTG
**Pr56**	Nedd8 (reverse) designed for ligation with plasmid EP222	ATAACTAATTACATGATGCGGCCCTCTAGACTAACCACCTCTTAGTGTTA

Table S4 (complementary for Figure 1).

H. sapiens **VS** ***S. cerevisiae***	**NEDD8 E1-activating enzyme** **UBA3** [*1]	**CSN5 with CSN5i-3** **CSN5 with CSN5i-3** [*2]
Sequence identity (%)	47%	27.07%
Coverage (%)	96%	44%
GMQE	0.78	0.18
QMEAN	-2.16	-2.85

[*1] Built on the human NEDD8-activating enzyme in complex with NEDD8 and MLN4924 crystal structure (PDB ID:3GZN, [11]). [*2] Built on PDB ID 5JOG [12].

Supplemental Alignment

Sequences with a high confidence alignment -

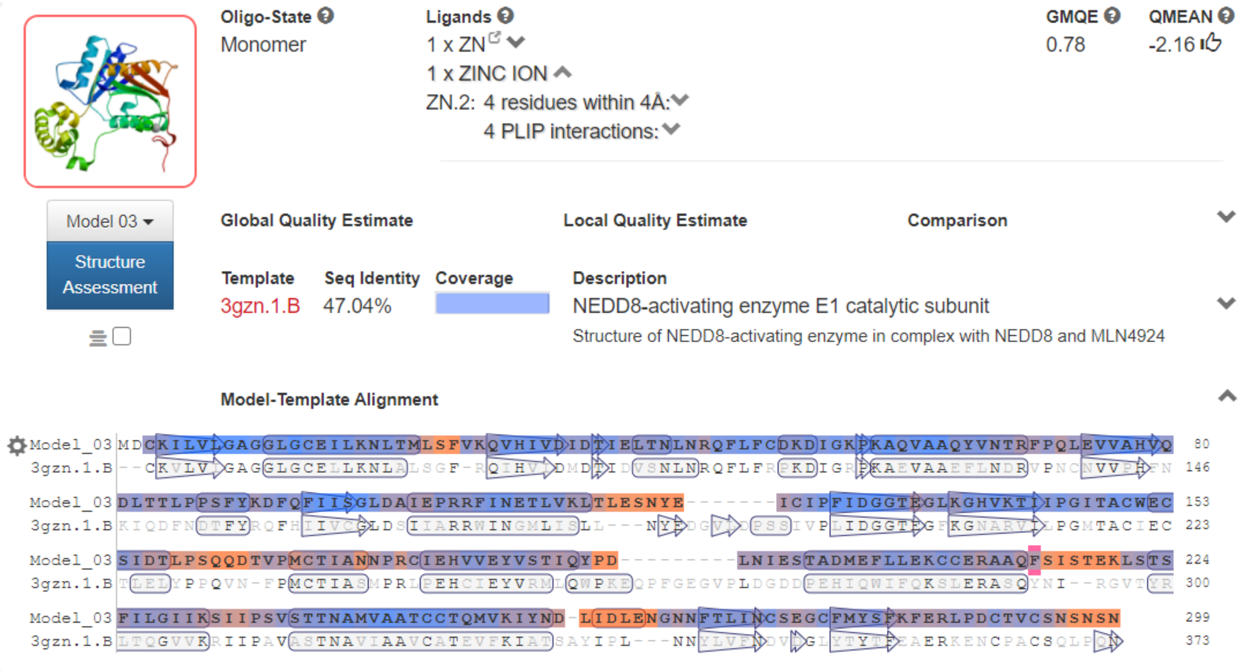

For figure 1B: *S. cerevisiae* Uba3, built on the structure of NEDD8-activating enzyme in complex with NEDD8 and MLN4924 (PDB ID: 3GZN).

GMQE = 0.78. **QMEAN** = -2.16. **Sequence identity** = 47.04%. **Coverage** = 96%.

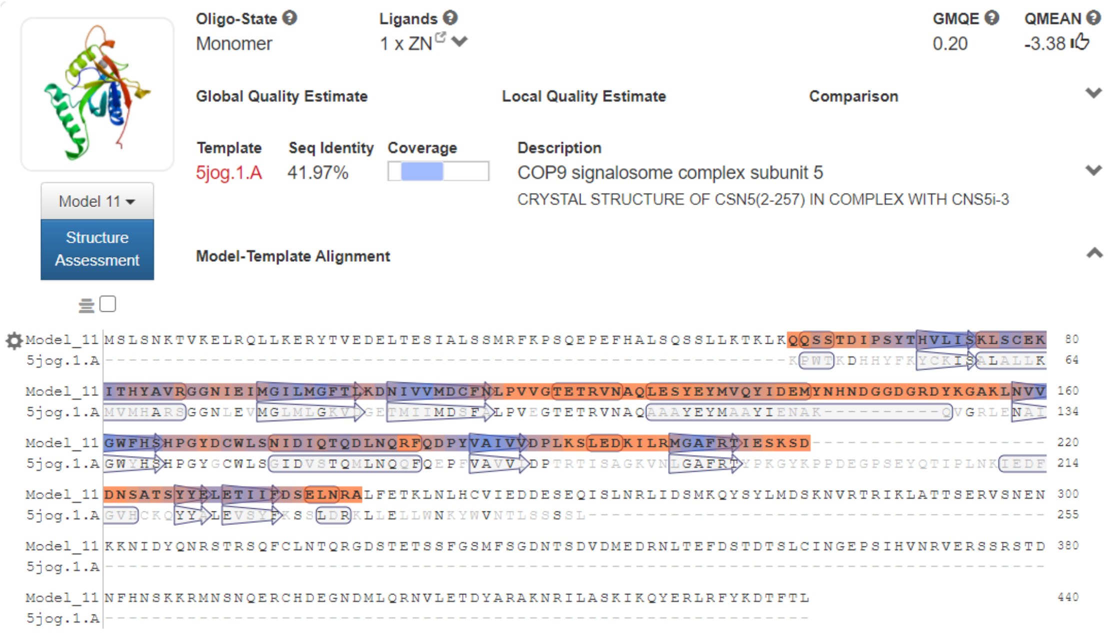

For figure 1C: *S. cerevisiae* Csn5, based on the structure of the human Csn5 in complex with CSN5i-3 (PDB ID: 5JOG) GMQE = 0.20, QMEAN = -3.38. Seq identity = 41.97%. Coverage = 44%.

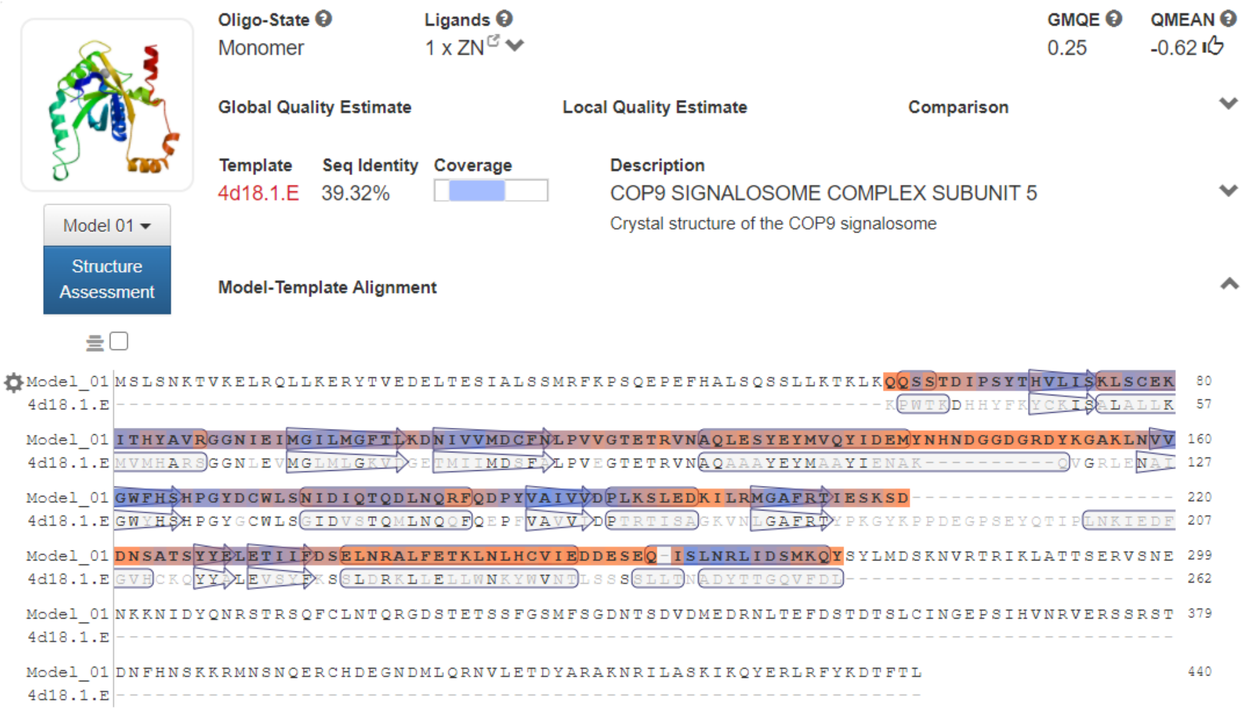

For figures 2C (left) , 2D and S1A: *S. cerevisiae* Csn5, built on the template of the human CSN5 crystal structure (PDB ID: 4D18).

GMQE = 0.25. **QMEAN** = -0.62. **Sequence identity** = 39.32%. **Coverage** = 47%.

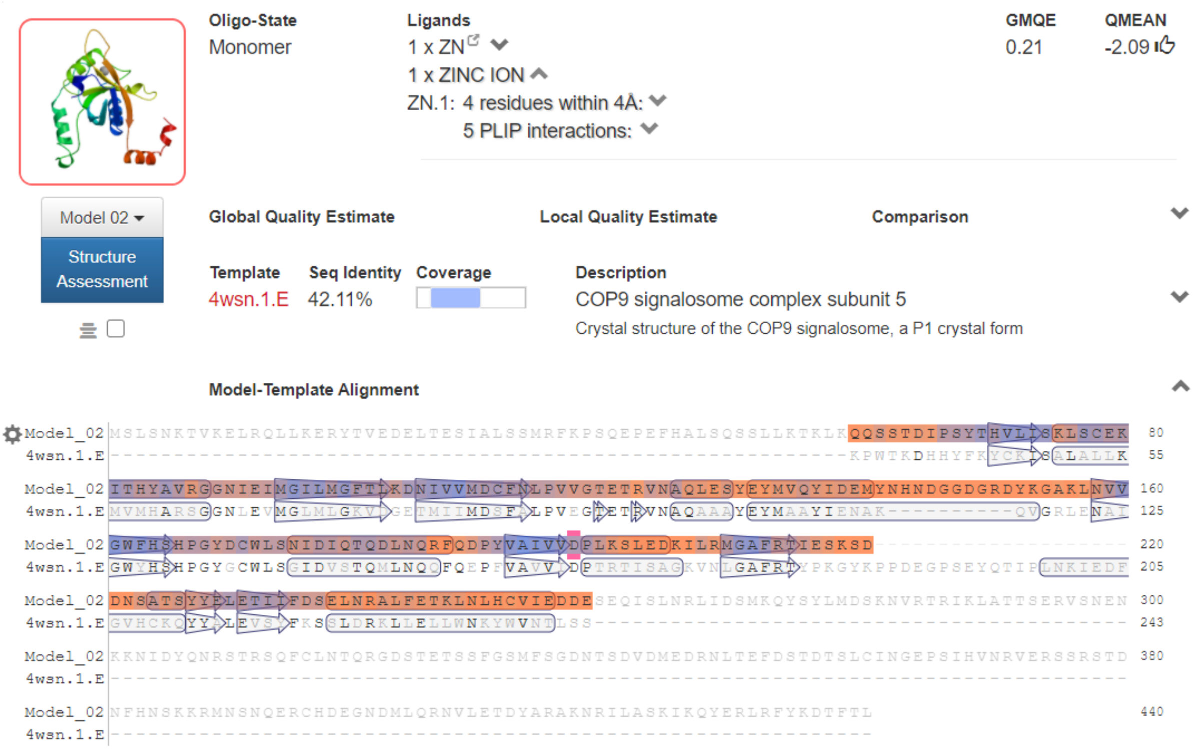

For figure S1: *S. cerevisiae* Csn5, built on the template of the human CSN5 crystal structure (PDB ID: 4WSN).

GMQE = 0.21. **QMEAN** = -2.09. **Sequence identity** = 42.11%. **Coverage** = 43%.

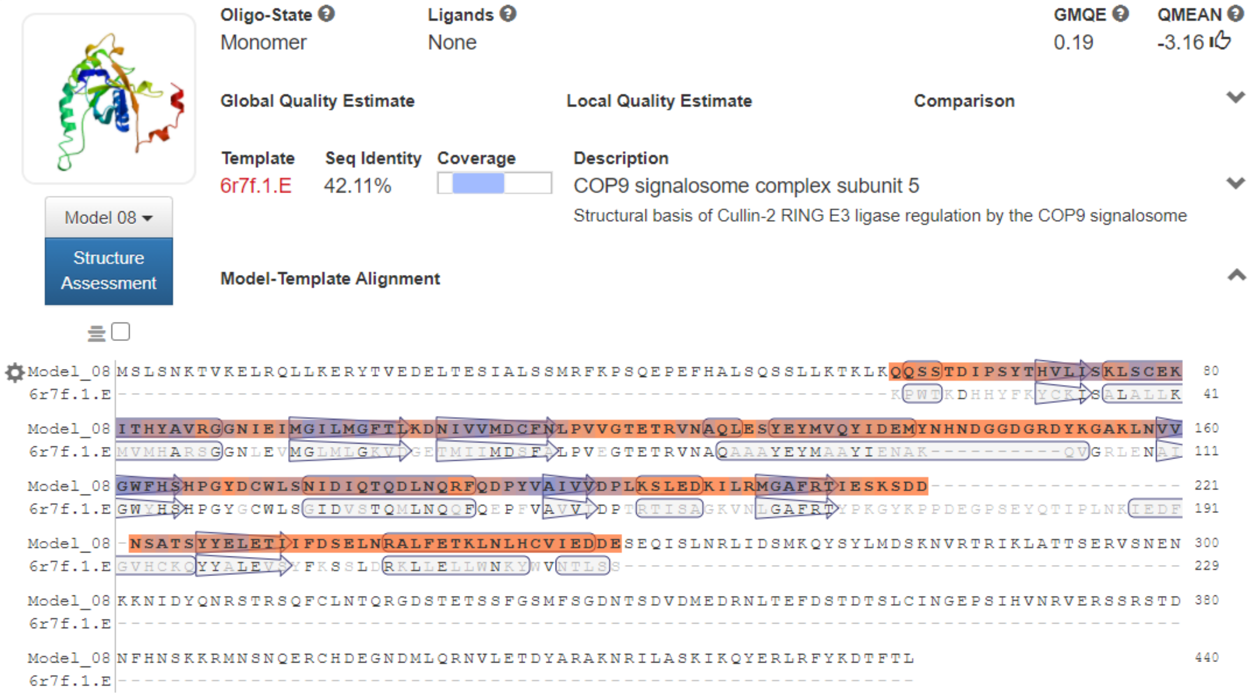

<u>For figure S1</u>: *S. cerevisiae* Csn5, built on the template of the human CSN5 crystal structure (PDB ID: 6R7F).

GMQE = 0.19. **QMEAN** = -3.16. **Sequence identity** = 42.11%. **Coverage** = 43%.

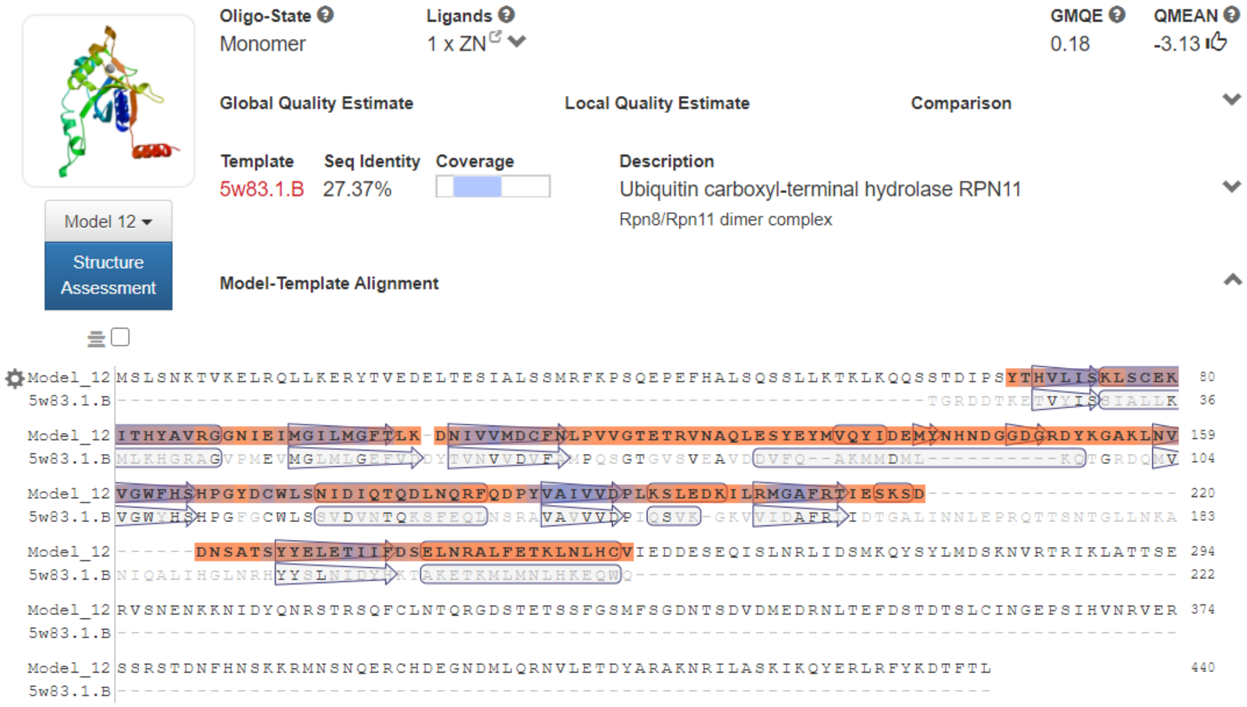

For figure S1: *S. cerevisiae* Csn5, built on the template of the human CSN5 crystal structure (PDB ID: 5W83).

GMQE = 0.18. **QMEAN** = -3.13. **Sequence identity** = 27.37%. **Coverage** = 43%.

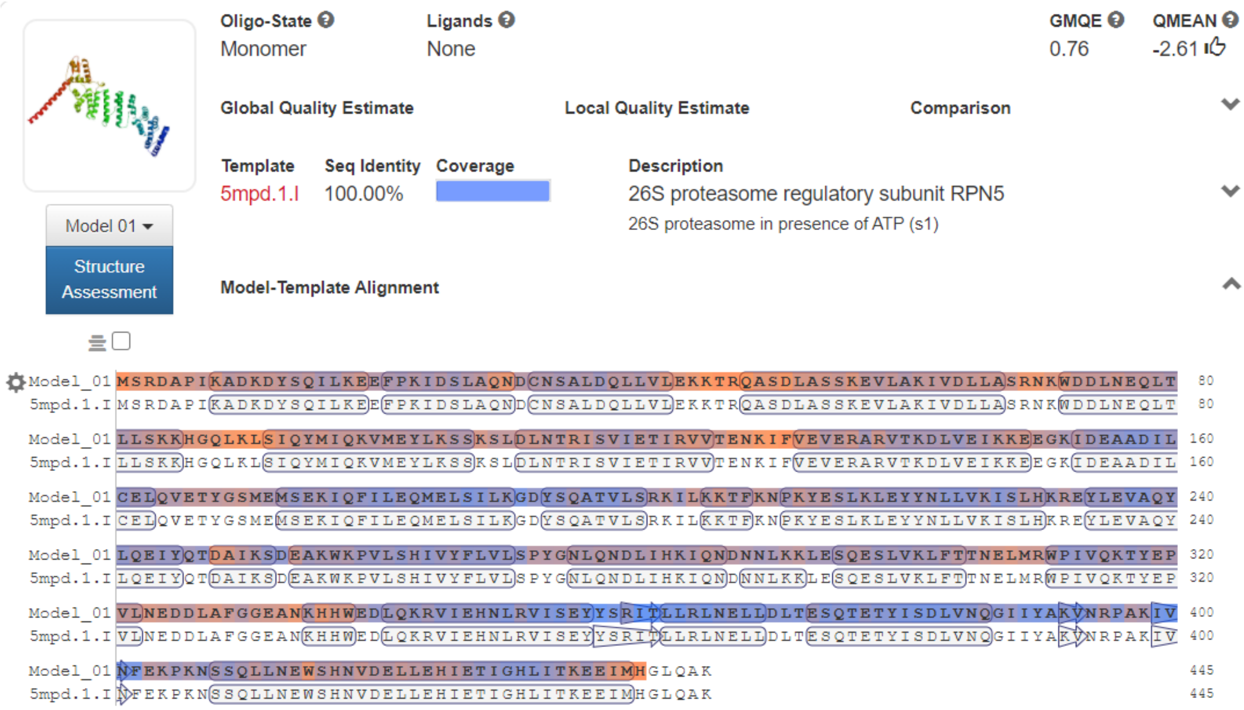

For figure 2D: *S. cerevisiae* Rpn5, built on the template of the *S. cerevisiae* 26S proteasome Rpn5 crystal structure (PDB ID: 5MPD).

GMQE = 0.76. **QMEAN** = -2.61. **Sequence identity** = 100%. **Coverage** = 100%.

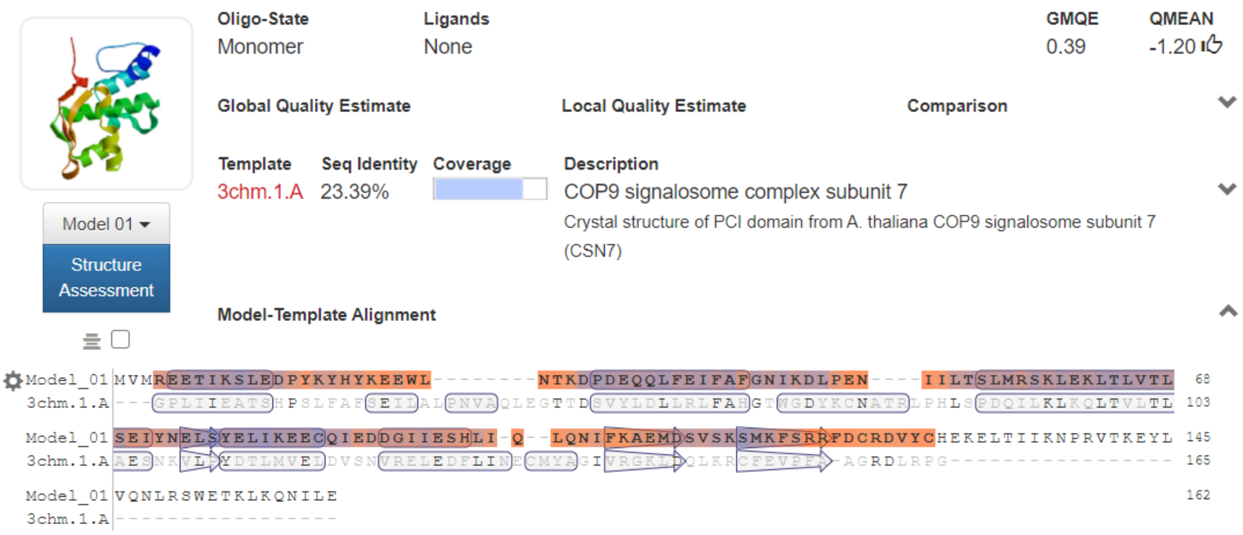

For figure 2D: *S. cerevisiae* Csn9, built on the template of the *A. thaliana* CSN7 crystal structure (PDB ID: 3CHM).

GMQE = 0.39. **QMEAN** = -1.2. **Sequence identity** = 23.39%. **Coverage** = 77%.

In three cases the QMEAN parameters were < (-4). For these cases Phyre2 confidence was calculated as well:

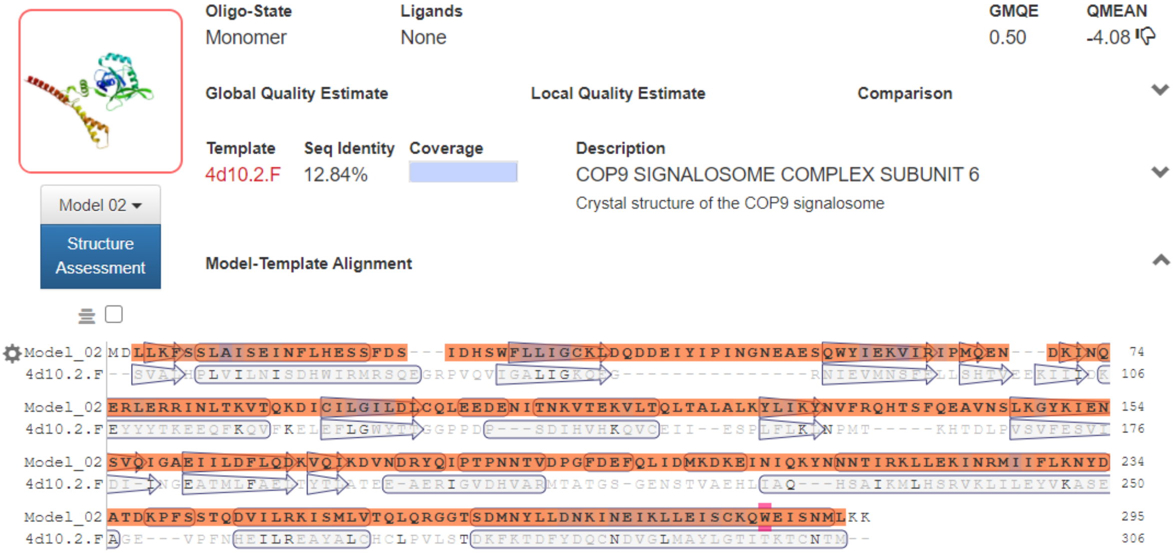

<u>For figure 2C (left), 2D</u>: *S. cerevisiae* Csi1, built on the template of the human CSN6 crystal structure (PDB ID: 4D10).

GMQE = 0.5. **QMEAN** = -4.08 (Low quality homology model). **Sequence identity** = 12.84%. **Coverage** = 87%.

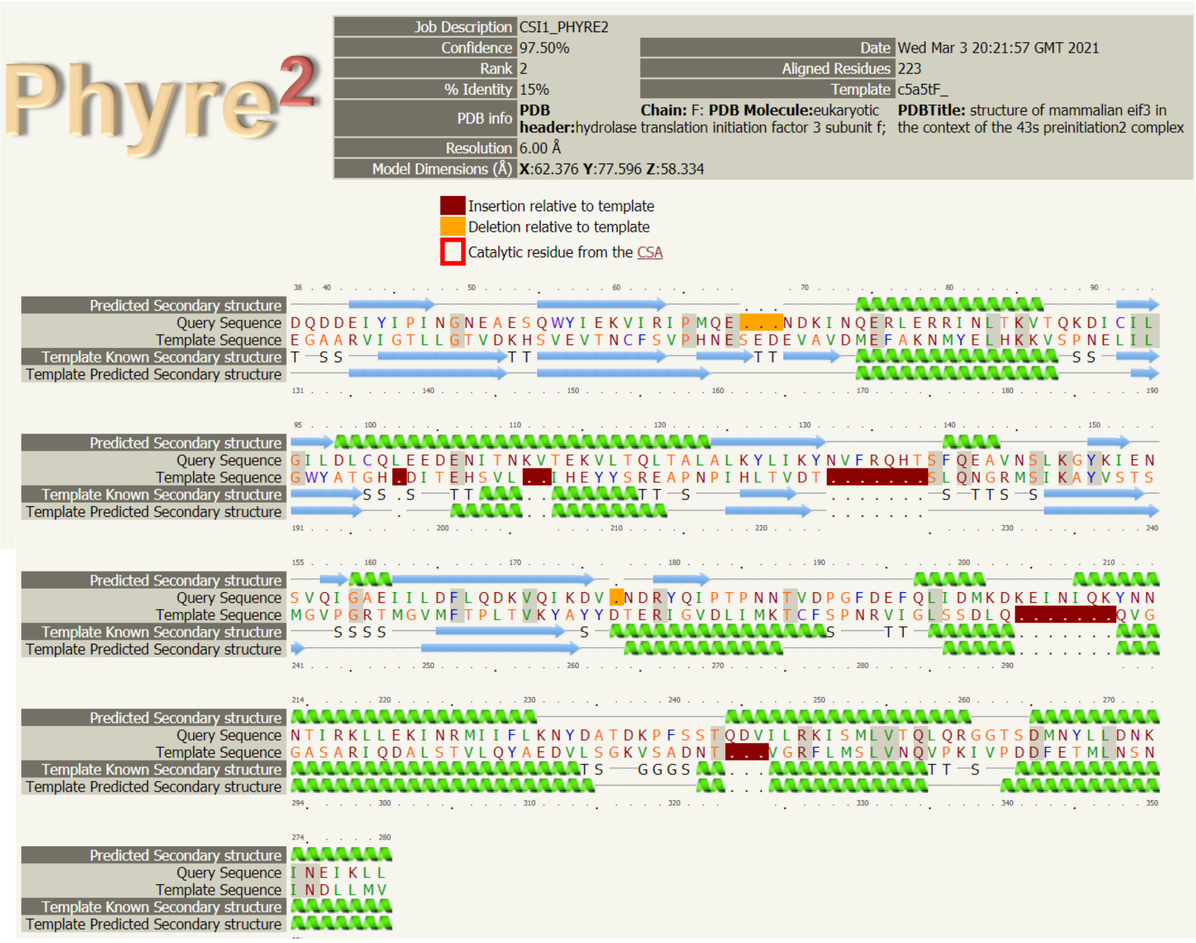

For figure 2C (left), 2D: *S. cerevisiae* Csi1, built on the template of the *Oryctolagus cuniculus* eIF3F crystal structure (PDB ID: 5A5T).

Confidence = 97.5%. **Sequence identity** = 15%. **Coverage** = 82%.

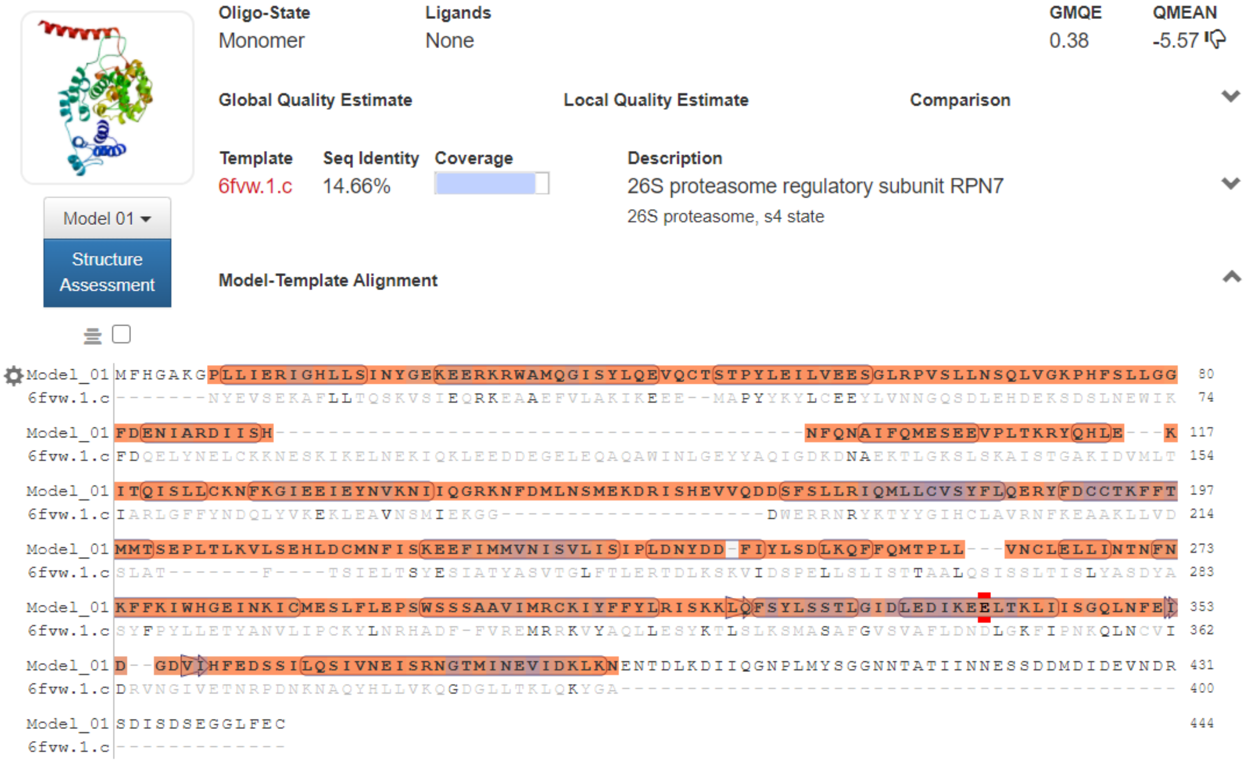

For table 1: *S. cerevisiae* Csn11, built on the template of the *S. cerevisiae* CSN1 crystal structure (PDB ID: 6FVW).

GMQE = 0.38. **QMEAN** = -5.57 (Low quality homology model). **Sequence identity** = 14.66%. **Coverage** = 78%.

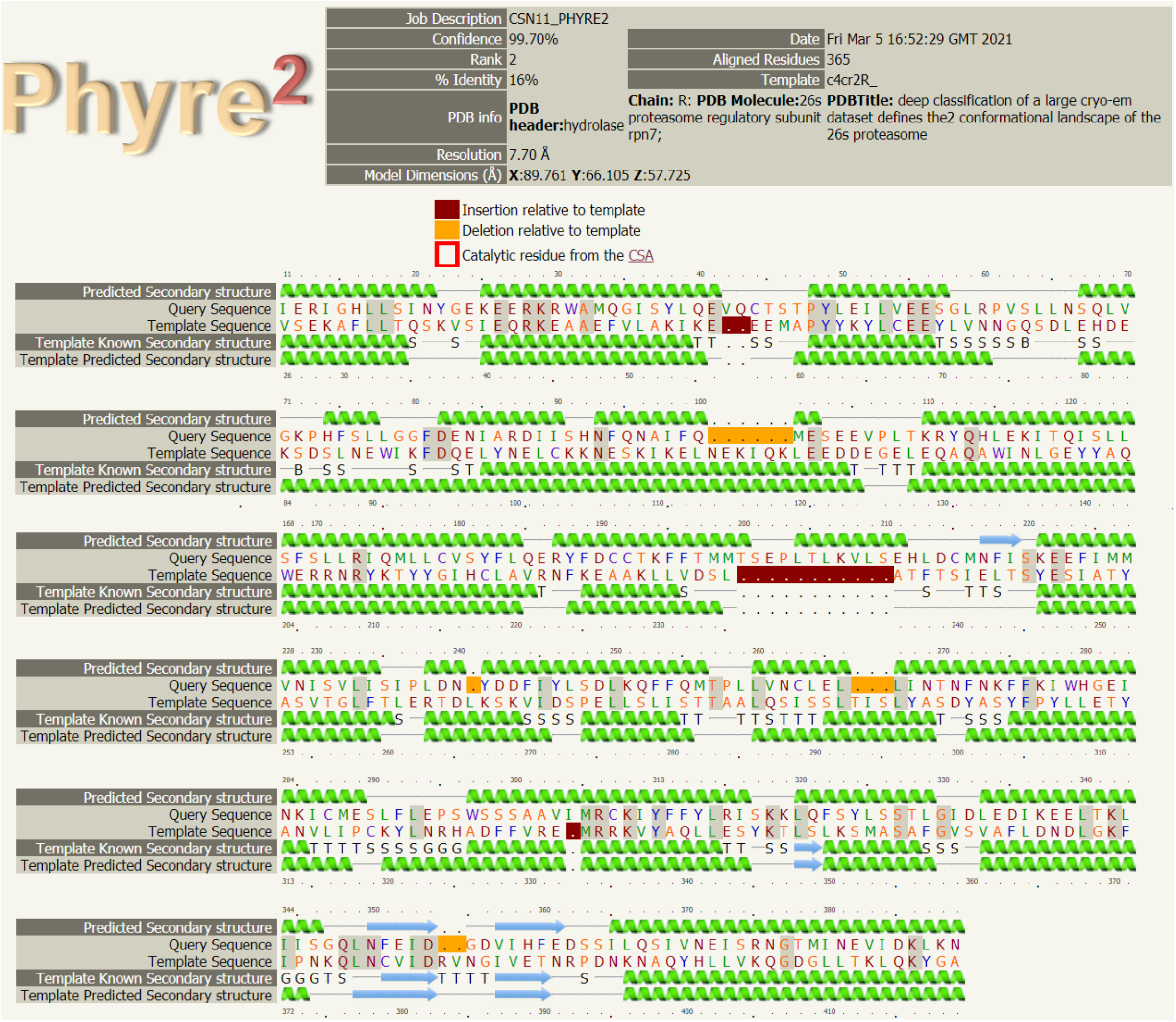

For table 2, 3: *S. cerevisiae* Csn11, built on the template of the *S. cerevisiae* Rpn7 crystal structure (PDB ID: 4CR2).

Confidence = 99.7%. **Sequence identity** = 16%. **Coverage** = 85%.

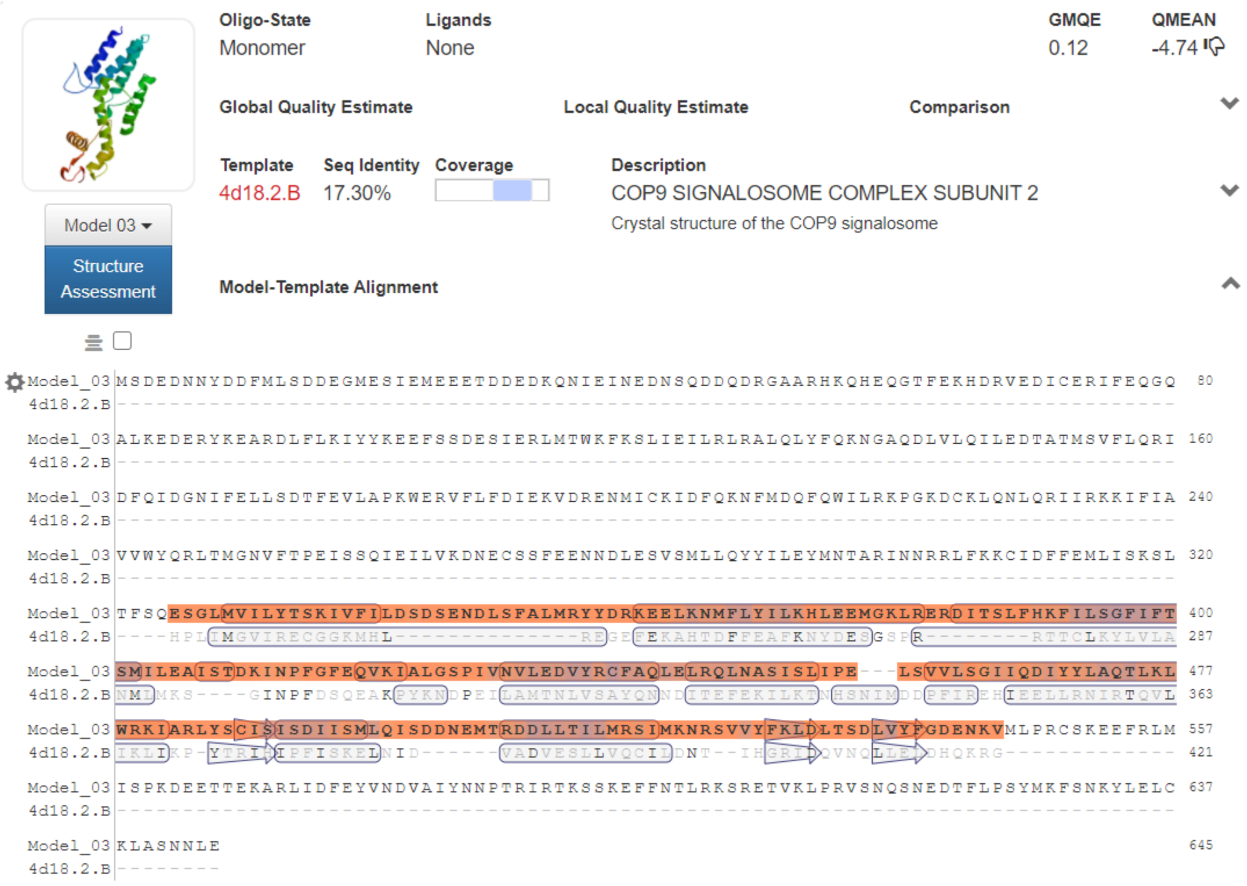

For table 1: *S. cerevisiae* Csn10, built on the template of the human CSN2 crystal structure (PDB ID: 4D18).

GMQE = 0.12 = -4.74 (Low quality homology model). **Sequence identity** = 17.3%. **Coverage** = 29%.

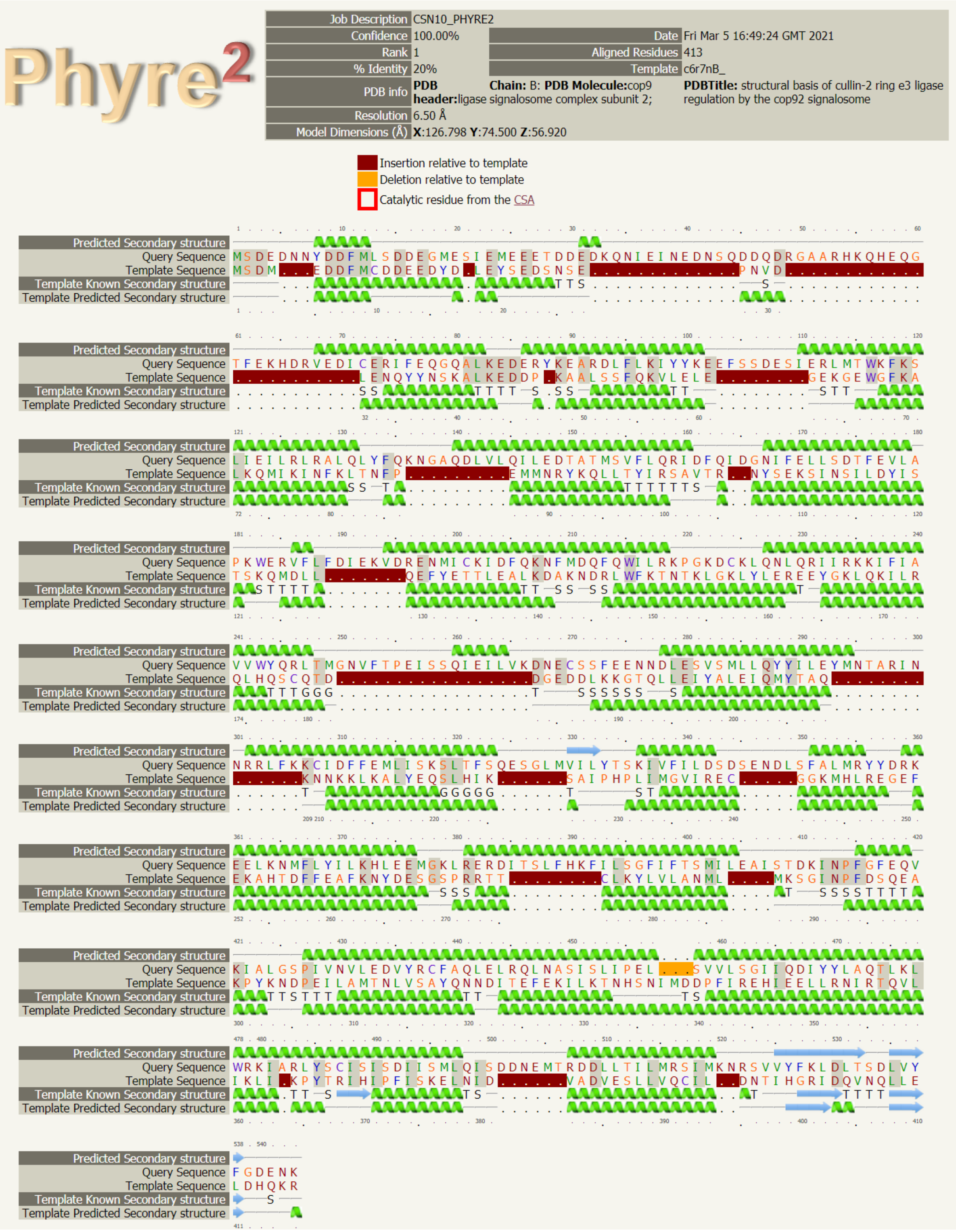

For tables 2, 3: *S. cerevisiae* Csn10, built on the template of the human Cullin-2 RING E3 ligase (CSN2) crystal structure (PDB ID: 6R7N).

Confidence = 100%. **Sequence identity** = 20%. **Coverage** = 84%.

Bibliography

1. Lingaraju, G.M., et al., *Crystal structure of the human COP9 signalosome.* Nature, 2014. **512**(7513): p. 161-5.
2. Eisele, M.R., et al., *Expanded Coverage of the 26S Proteasome Conformational Landscape Reveals Mechanisms of Peptidase Gating.* Cell Rep, 2018. **24**(5): p. 1301-1315 e5.
3. Wehmer, M., et al., *Structural insights into the functional cycle of the ATPase module of the 26S proteasome.* Proc Natl Acad Sci U S A, 2017. **114**(6): p. 1305-1310.
4. Dessau, M., et al., *The Arabidopsis COP9 signalosome subunit 7 is a model PCI domain protein with subdomains involved in COP9 signalosome assembly.* Plant Cell, 2008. **20**(10): p. 2815-34.
5. Biasini, M., et al., *SWISS-MODEL: modelling protein tertiary and quaternary structure using evolutionary information.* Nucleic Acids Res, 2014. **42**(Web Server issue): p. W252-8.
6. Benkert, P., S.C. Tosatto, and D. Schomburg, *QMEAN: A comprehensive scoring function for model quality assessment.* Proteins, 2008. **71**(1): p. 261-77.
7. Bramasole, L., et al., *Proteasome lid bridges mitochondrial stress with Cdc53/Cullin1 NEDDylation status.* Redox Biol, 2019. **20**: p. 533-543.
8. Sinha, A., et al., *The COP9 signalosome mediates the Spt23 regulated fatty acid desaturation and ergosterol biosynthesis.* FASEB J, 2020.
9. Yu, Z., et al., *Dual function of Rpn5 in two PCI complexes, the 26S proteasome and COP9 signalosome.* Mol Biol Cell, 2011. **22**(7): p. 911-20.
10. Ziv, I., et al., *A perturbed ubiquitin landscape distinguishes between ubiquitin in trafficking and in proteolysis.* Mol Cell Proteomics, 2011. **10**(5): p. M111 009753.
11. Brownell, J.E., et al., *Substrate-assisted inhibition of ubiquitin-like protein-activating enzymes: the NEDD8 E1 inhibitor MLN4924 forms a NEDD8-AMP mimetic in situ.* Mol Cell, 2010. **37**(1): p. 102-11.
12. Schlierf, A., et al., *Targeted inhibition of the COP9 signalosome for treatment of cancer.* Nat Commun, 2016. **7**: p. 13166.
